# Artificial Tear Instillation-Induced Changes in Corneal Topography

**DOI:** 10.3390/bioengineering11020121

**Published:** 2024-01-26

**Authors:** Yunjin Lee, Tae Hun Kim, Hae Jung Paik, Dong Hyun Kim

**Affiliations:** 1 Department of Ophthalmology, Gachon University College of Medicine, Gil Medical Center, 21, Namdong-daero 774beon-gil, Namdong-gu, Incheon 21565, Republic of Korea; yunjin860823@naver.com (Y.L.); hjpaik@gilhospital.com (H.J.P.); 2 Department of Ophthalmology, Korea University College of Medicine, 73, Goryeodae-ro, Seongbuk-gu, Seoul 02841, Republic of Korea

**Keywords:** corneal topography, keratometry, astigmatism, artificial tear

## Abstract

This study aims to compare changes of corneal topography (Galilei G4) before and after the instillation of artificial tears in patients with dry eye disease (DED). Corneal topography was performed in patients 1 min before and after artificial tear instillation. Two types of artificial tears were used: 1% polysorbate 80 (PSB) and 0.5% carboxymethylcellulose (CMC). Of 135 patients, PSB and CMC were instilled in 101 and 34 eyes, respectively. The average value of Sim K increased significantly after instillation (44.07 ± 2.26 diopter (D)) compared to before (43.90 ± 2.02 D, *p* = 0.006) the instillation of artificial tears. Mean Sim K astigmatism was statistically increased after PSB instillation (1.48 ± 2.17 D) compared to before instillation (1.31 ± 2.10 D, *p* = 0.049). An axis change of astigmatism 10° or more after artificial tear instillation was found in 51.9% of patients, and 30° or more in 20.0% of patients. Increased Sim K value and significant changes in the astigmatic axis in the corneal topography were observed after instillation of artificial tears in DED patients. PSB instillation had a greater effect on corneal keratometry values than CMC instillation.

## 1. Introduction

In modern cataract surgery, accurate biometric measurements are paramount for achieving the expected refraction [1]. Various formulas have been proposed for intraocular lens (IOL) power selection in cataract surgery [2], and values such as corneal refractive power (K), axial length, lens thickness, and anterior chamber depth are required, but the most important factor among them is the K value [3]. K values depend on the reflection of mires from the tear film; therefore, the stability of the tear film and ocular surface can affect the reliability of K measurements. 

Dry eye disease (DED), characterized by an unstable tear film with an irregular surface, is one of the most common ophthalmic diseases [4], mostly affecting people in modern society [5]. Artificial tears are commonly used in DED to lubricate the ocular surface and relieve symptoms such as burning, stinging, and gritty sensations. A significant variability in average K and anterior corneal astigmatism was observed in patients with DED [6]. Therefore, some studies recommend use of artificial tears, which can increase tear film stability, to obtain an accurate K value [6,7]. Additionally, optical aberrations associated with irregular tear films in patients with DED significantly improved after the use of artificial tears [8]. However, Röggla et al. revealed that when different concentrations of artificial tears were instilled prior to biometry measurement using IOLMaster500, a significant difference in K values was observed, especially in patients with DED [9]. They recommended that ocular biometric measurements be taken at least 5 min after the instillation of eye drops [9]. Similar studies have been conducted previously; however, studies comparing topographic findings before and after artificial tear instillation are limited.

Herein, we investigated the changes in topographic findings before and after artificial tear instillation in DED patients using a Galilei^®^ G4 Dual Scheimpflug Analyzer (Galilei) (Ziemer Ophthalmic Systems, Port, Switzerland). In addition, because the effects may differ depending on the components of the artificial tears, we used two types of artificial tears: 1% polysorbate 80 (PSB) and 0.5% carboxymethylcellulose (CMC). 

## 2. Methods

### 2.1. Study Design and Patients

This study was approved by the Institutional Review Board (IRB) of the Gachon University Gil Medical Center (IRB number: GAIRB2023-162) and informed consent was waived due to the retrospective nature of the study. The study followed the tenets of the Declaration of Helsinki.

We performed a retrospective analysis of patients who had been diagnosed with DED at the Gachon University Gil Medical Center between 1 March 2021 and 31 May 2021. Only mild to moderate dry eye patients were recruited in this study. The inclusion criteria for patients were as follows: (1) age 20 years or older; (2) those who had experienced DED related symptoms (such as dryness, foreign body sensation, or irritation) for at least 1 month; (3) one or multiple symptoms were involved: ocular dryness, burn and foreign body sensation, visual blur, or discomfort like night vision, reading, and so forth; and; (4) topographic measurements with Galilei before and 1 min after PSB or CMC instillation. Patients with the following conditions were excluded: presence of any uncontrolled systemic disease, presence of any severe ocular surface disease and/or corneal epithelial pathology, previous ocular trauma or surgery other than uncomplicated cataract surgery, history of cataract or refractive surgery within the last 6 months, contact lens wear, and continuous eye drop use (e.g., antibiotics, antivirals, steroids, glaucoma medications, lipid emulsion eye drops, and lubricant ointment) other than artificial tears. 

Two types of artificial tears were evaluated in this study: PSB and CMC (Table 1). Both types of artificial tears are commonly used for dry eyes. PSB acts as an emulsifier and helps stabilize the lipid layer of the tear film, while CMC is an anionic cellulose polymer that is beneficial for increasing tear retention time [10]. 

Galilei^®^ G4 Dual Scheimpflug Analyzer (Galilei) (Ziemer Ophthalmic Systems, Port, Switzerland) combines a Placido disc ring to evaluate the anterior corneal surface with a rotational scan of dual Scheimpflug slit images that compensates for decentrations due to eye movement. The Galilei G4 has a red LED that serves as a fixation target and can be moved in 0.25 D steps from −20 D to +20 D. The Galilei was calibrated daily before the first measurement, following the manufacturer’s instructions. Every time we examined the patient, the room was constantly illuminated under 10 lx, as measured using a light meter (LX-1102; Lutron, Taipei, Taiwan). Measurements were performed by a single specialist using a standard methodology. During measurement, all patients were asked to keep their chin and forehead in position and look at the fixation light. Complete eye blinking was required before each automatic capture. The measurement was accepted for analysis if image quality status showed “OK” on the device screen. Otherwise, the measurements were repeated until a high-quality image was obtained. The device was realigned to its default position before subsequent measurements. Because of changes in the tear film, which can affect Placido disc measurements and the quality of corneal images, the examiner waited 3 s between the blink and the acquisition of each image. After each measurement, subjects were allowed to sit back for 30 s to allow for realignment of the device. Subjects were instructed to blink prior to each measurement. We only included scans that met the manufacturer’s minimum acceptable quality factors in the Galilei G4. The internal software calculates the percentages for the quality parameters, which include motion compensation, placido, Scheimpflug, and motion distance; the minimum values were 85%, 85%, 90%, and 70%, respectively.

The measured parameters included average simulated keratometry (Sim K); astigmatism of Sim K; angle of steep astigmatism; central, mid, and peripheral K of anterior axial curvature; total corneal power (TCP); astigmatism of TCP, and Kappa distance. The Keratoconus Prediction Index (KPI) was also used to evaluate corneal irregularity. Tear breakup time (TBUT) and corneal ocular surface staining (OSS) were used as dry eye parameters. To measure Kappa distance, the Galilei G4 automatically measures the distance between the center of the pupil and the center of the reflection of the four Purkinje points, which corresponds to the first Purkinje reflex in the cornea of four light points included in the Galilei G4, with a measurement resolution of 0.01 mm.

### 2.2. Statistical Analysis

Statistical analyses were performed using SPSS Complex Samples procedures (PASW Statistics for Windows, version 18.0; SPSS Inc., New York, NY, USA). A paired *t*-test was performed to compare the results before and after the instillation of artificial tears. The chi-square test or independent *t*-test was applied to the analyses of the two artificial tear groups. A *p*-value < 0.05 indicated statistical significance. The results are presented as the means ± standard deviations (SDs) unless otherwise indicated. The double-plot angle was used to analyze the astigmatism before and after the instillation of artificial tears. 

## 3. Results

This study included 135 patients, comprising 65 men and 70 women, with a mean age of 57.2 ± 16.6 years. Among them, PSB and CMC were used in 101 patients (48 men and 53 women) and 34 patients (17 men and 17 women), respectively. Table 2 shows the baseline characteristics of the enrolled patients. Age, sex, and other parameters of corneal topography and dry eyes before instillation of artificial tears were not significantly different between the two groups (all *p* > 0.05). 

Table 3 shows a comparison of corneal topography values before and after artificial tear instillation (PSB or CMC). First, changes in topographic values before and after artificial tear instillation were evaluated in all patients. The mean simulated keratometry (Sim K) (43.90 ± 2.02–44.07 ± 2.26, *p* = 0.006) and mid K of anterior axial curvature (43.32 ± 1.84–43.42 ± 1.88, *p* = 0.027) were significantly increased after instillation of artificial tears compared to before instillation. Statistically significant differences were not observed in other parameters (astigmatism of Sim K, steep axis of astigmatism, central, mid, and peripheral K of anterior axial curvature, mean total corneal power, astigmatism of total corneal power, kappa distance, and KPI). Although the steep axis of astigmatism before and after instillation of artificial tears was not statistically different, the astigmatic axis changed by more than 10° in 70 patients (51.9%) and by more than 30° in 27 patients (20.0%). Next, the groups were divided according to the type of artificial tear, and the changes were examined. The average Sim K increased in the PSB instillation group (43.86 ± 2.04–44.04 ± 2.36, *p* = 0.012), whereas no difference was observed in the CMC instillation group. KPI (%) was significantly decreased in PSB group (19.70 ± 20.67–16.70 ± 16.12, *p* = 0.028), however there was no change in CMC group.

**Figure 1:**
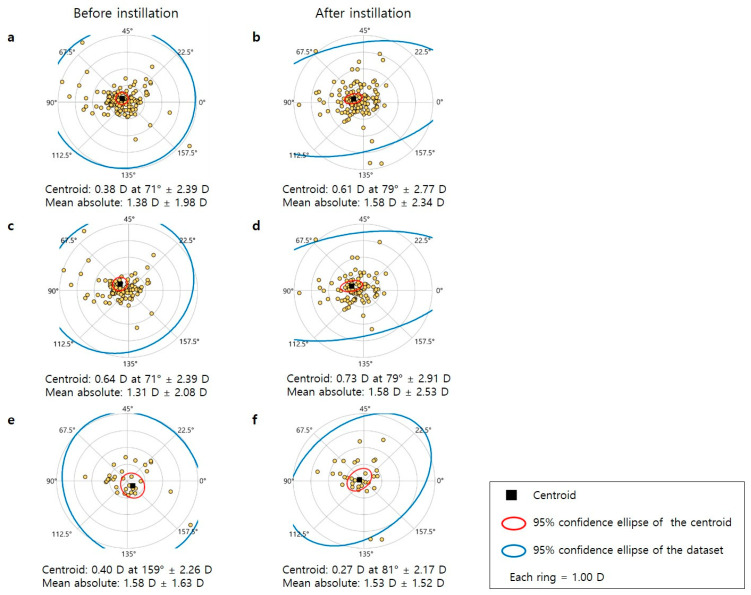
Double angle plot for astigmatism before and after instillation of artificial tears. After the instillation of artificial tears, the centroid and mean absolute astigmatism in the corneal plane increased slightly ((**a**): before, (**b**): after). PSB group exhibited this tendency ((**c**): before, (**d**): after); however, it was difficult to confirm in the CMC group ((**e**): before, (**f**): after).

Figure 1 shows a double-angle plot of astigmatism before and after artificial tear instillation. After the instillation of artificial tears, the centroid and mean absolute astigmatism on the corneal plane were slightly increased (0.38 ± 2.39–0.61 ± 2.77, 1.38 ± 1.98–1.58 ± 2.34, respectively, Figure 1a,b). This tendency was same in the PSB group (0.64 ± 2.39–0.73 ± 2.91, 1.31 ± 2.08–1.58 ± 2.53, respectively, Figure 1c,d), but was difficult to confirm in the CMC group (0.40 ± 2.26–0.27 ± 2.17, 1.58 ± 1.63–1.53 ± 1.52, respectively, Figure 1e,f). 

**Figure 2:**
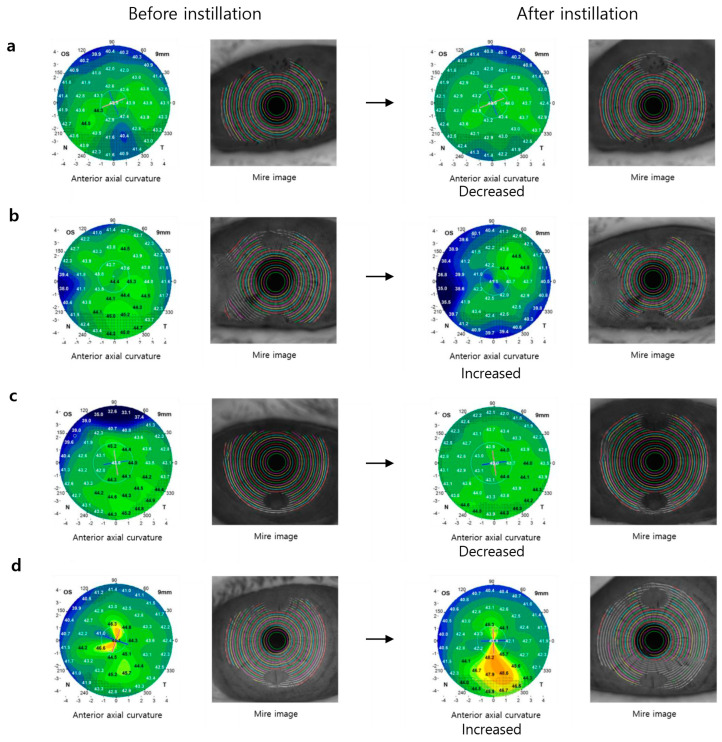
Representative photographs of the anterior axial curvature map and mire ring before and after instillation of artificial tears. In some cases, the difference in K value decreased after instillation of artificial tears (**a**,**c**), and in other cases, it increased (**b**,**d**). ((**a**,**b**): CMC, (**c**,**d**): PSB).

Representative images with significant differences (increase or decrease) in the K value of the anterior axial curvature map before and after the instillation of artificial tears are shown in Figure 2. In some cases, the difference in K values decreased after instillation of artificial tears (Figure 2a,c), and in other cases, it increased (Figure 2b,d). 

## 4. Discussion

In this study, we assessed the impact of artificial tears on corneal topography by comparing corneal topography values before and 1 min after instillation of artificial tears in patients with DED. Although consistent results were not obtained between the two types of artificial tears in our study, an increase in the average Sim K and an alteration in the axis of astigmatism were confirmed after the instillation of artificial tears. Overall, PSB instillation resulted in greater changes in topographic findings than did CMC instillation in DED patients. After instilling PSB or CMC eye drops, the topographic findings and corneal mires in the cornea changed more regularly in some cases and more irregularly in others.

The effects of artificial tears on ocular surface regularity and visual functions vary; some report improvement, while others do not even after the instillation of artificial tears. Artificial tears improved the quality of the obtained images [11,12]. Mico et al. demonstrated that this was due to a significant reduction in the total, sphere-like, and comma-like aberrations after the application of artificial tears [8]. However, in another study, the authors recommended that topography be performed before artificial tear instillation as a baseline image, noting that the effects of artificial tears differed between regular and irregular corneas that had undergone penetrating keratoplasty [13]. This study concluded that the addition of artificial tears to eyes without abnormalities worsened symmetry, whereas asymmetric and irregular-surfaced eyes became more regular after artificial tear application. Consistent with our results, Veronika et al. reported that both keratometry values and astigmatism showed significantly increased variability after instillation of both low- and high-viscosity eye drops, especially in patients with DED [9]. However, these effects were no longer statistically significant 5 min after instillation.

This study reported an elevated mean Sim K after instillation. When patients were divided into the PSB and CMC groups, the mean Sim K increased only in the PSB group. PSB altered the Sim K value as it improves the regularity of the cornea because the KPI value is reduced after instillation; however, no changes in the ocular surface were observed after CMC instillation as it does not substantially change the KPI and Sim K values. Alternatively, CMC does not have an effect on the corneal surface lasting > 1 min; however, PSB may have a slightly longer effect on the corneal surface [14]. This could be attributed to differences in the composition of the two artificial tears. CMC, a cellulose derivative, increases the viscosity of tears and is beneficial in increasing tear retention time [15]. In contrast, PSB, which acts as an emulsifier, stabilizes the lipid layer of the tear film by reducing tear film evaporation and effective in DED [14,16,17]. Therefore, differences in ingredients may have contributed to the differences in the results. Further research is needed to determine how the composition of the artificial tears affects the results of corneal topography or ocular biometry measurements. In addition, if artificial tears are used prior to ocular biometry measurements, a relevant artificial tear should be selected to minimize measurement variability.

In the analysis of the central, mid (paracentral), and peripheral zones of the anterior axial curvature, the mid-K value was significantly different before and after artificial tear instillation. In the Galilei topography, the central, mid, and peripheral zones measured K values in the 0–4, 4–7, and 7–10 mm diameters, respectively. Although the results are conflicting and the optimal diameter region for measurement is inconclusive owing to inconsistencies in previous results and variability in study designs [18,19,20,21,22], some studies suggest that the paracentral zone is more accurate than those at smaller diameter zones for astigmatism prediction in cataract surgery [18,20]. Therefore, ascertaining the fact that the mid K value of anterior axial curvature can change after artificial tear instillation in DED is imperative. However, the deviations of these values were of limited clinical significance including ≤0.5 D, so there may be a different interpretation but it is necessary to be aware of the differences.

Several investigators have assessed corneal astigmatism induced by tear instillation [23,24]. Some studies reported a large change of approximately 0.7 D after 1 min of tear instillation [24], and another study reported a change of less than 0.3 D [23]. In our study, no statistically significant differences in the degree of astigmatism were identified before and after artificial tear instillation; however, significant changes were identified in the astigmatism axis. More than 50% of enrolled patients showed a change in the axis of astigmatism by 10° or more, which was similar when the patients were divided into PSB and CMC groups. Astigmatic axis changes > 30° were observed in 20% of all patients (21.8% in the PSB instillation group and 14.7% in the CMC instillation group). Axis evaluation plays a significant role in toric IOL in cataract surgery [3,25]. As already known, a toric IOL with 10 degrees of misalignment has a 34% reduction in astigmatism prevention effect and an IOL with 30 degrees of on-axis misalignment has no astigmatism correction effect [3,25]. Additionally, refractive correction surgery [26] as well as contact lens fitting are also influenced according to the axis of astigmatism. Therefore, considering that the axis of astigmatism may change in topography after artificial tear instillation, caution is needed when patients using artificial tears are examined for cataracts, refractive surgery, or contact lens fitting.

Our study had some limitations. First, because of the retrospective nature of the study, only two types of artificial tears, CMC and PSB, were compared, and only the short-term effects of the artificial tears were evaluated. As many different types of artificial tears are currently available, their effectiveness needs to be studied in the future. In addition, if time-dependent changes were observed, the PSB results may have been different. The effect of artificial tears over time has been studied previously, and the effect resolved after 3–5 min [9,23]. In our study, the effect was evaluated after 1 min, and no differences were observed for CMC, whereas PSB exhibited deviations with limited clinical significance, including ≤0.5 D. This needs to be considered when applying our results to clinical practice. Second, the sample size of the CMC group was relatively smaller than that of the PSB group. Although the same cohort size was investigated during the study period, only 34 patients satisfied the inclusion criteria for the CMC group. Third, only patients with DED were reviewed. Artificial tears can affect normal, diseased, and postoperative corneas differently [13]. One study reported different effect of artificial tears between patients with and without punctate epitheliopathy [27]. Therefore, follow-up studies involving irregular ocular surfaces in various clinical settings are required. Fourth, changes of tear film thickness may affect the measurements and variations, however those were not evaluated. Fifth, continuous changes of the topographic findings after the instillation of artificial tear were not assessed. Nevertheless, our study has strength in that we observed topographic changes with two types of artificial tear instillation in real-world practice, and several values from topography, such as central, mid, peripheral K, kappa distance, and KPI (%), were compared between pre- and post-instillation of artificial tears. In a clinical situation, patients with DED may put artificial tears in their eyes immediately before topographic examination. There are also cases where topographic test results are not properly obtained due to ocular surface irregularity caused by dryness. In this case, the examiner may instill a drop of artificial tears into the patient’s eye and perform the test. As can be seen from the results of our study, test results may differ before and 1 min after artificial tears are instilled in DED patients. Therefore, examinees and physicians should be aware of this fact. According to the Preoperative Treatment Algorithm for Ocular Surface Diseases of the Clinical Committee of the American Society of Cataract and Refractive Surgery, treatment of DED prior to cataract or refractive surgery can optimize surgical outcomes and patient satisfaction [28]. DED is one of the most influential factors to increase the variability of preoperative corneal power measurement, leading to inaccurate IOL power prediction or unsatisfactory refractive surgery outcomes [28,29,30]. Therefore, it is important to treat DED sufficiently before cataract or refractive surgery to ensure that the ocular surface is healthy enough to avoid the need for artificial tears in the ocular biometry measurements. If it is necessary to use artificial tears prior to ocular biometry, CMC artificial tear seems to be more appropriate as it has less impact on the variability of the measurements and it is recommended to measure at least 5 min after instillation.

There was considerable variability in keratometric values and astigmatism axis in patients with DED 1 min after instillation of PSB and CMC artificial tears. In addition, a steepening of the mean sim K value was observed 1 min after PSB instillation in DED patients. Further research is needed on how to minimize the variability of ocular biometry and increase the accuracy of the measurements.

## Figures and Tables

**Table 1:** Main features of the preservative-free eye lubricants used in this study.

Product	Refresh Plus^®^ Lubricant Eye Drops	Revita^®^ Eye Drops
Active ingredient	0.5% sodium carboxymethyl cellulose	1.0% polysorbate 80
Other ingredients	Sodium chloride, sodium lactate solution, potassium chloride, calcium chloride hydrate, magnesium chloride hydrate, hydrochloric acid, sodium hydroxide, purified water	Sodium chloride, D-mannitol, retinyl palmitate, citric acid, sodium citrate hydrate, disodium edetate hydrate, sodium hydroxide, water for injection
Preservative	None	None
Manufacturer	Allergan	Eden pharma
Volume (mL)	0.8	0.4
Use	The instillation of 1 drop of artificial tear	The instillation of 1 drop of artificial tear

**Table 2:** Baseline demographics and clinical characteristics.

	All (n = 135)	PSB (n = 101)	CMC (n = 34)	*p* Value
Age (years)	57.2 ± 16.6	58.5 ± 16.0	53.4 ± 17.9	0.125
Sex (M/F)	65/75	48/53	17/17	0.803 ^†^
Laterality (R/L)	84/51	67/34	17/17	0.089 ^†^
Steep Sim K (D)	44.59 ± 2.48	44.52 ± 2.56	44.79 ± 2.24	0.576
Flat Sim K (D)	43.21 ± 2.00	43.21 ± 1.99	43.21 ± 2.02	0.987
Mean Sim K (D)	43.90 ± 2.02	43.86 ± 2.04	44.00 ± 1.97	0.725
Sim Kastig (D)	1.38 ± 2.00	1.31 ± 2.10	1.58 ± 1.65	0.496
Axis of Sim Kastig (°)	80.95	77.87	90.09	0.216
KPI (%)	19.27 ± 21.1	19.7 ± 20.7	18.0 ± 22.4	0.679
Kappa distance (mm)	0.37 ± 0.34	0.38 ± 0.37	0.34 ± 0.21	0.560
TBUT (sec)	5.14 ± 1.07	5.02 ± 1.16	5.50 ± 0.62	0.199
Cornea OSS (points)	0.48 ± 0.84	0.52 ± 0.87	0.36 ± 0.74	0.359

Values are presented as mean ± standard deviation. PSB: Polysorbate 80; CMC: 0.5% carboxymethylcellulose; Sim K: simulated keratometry; Sim Kastig: astigmatism between flat and steep; D: diopter; KPI: Keratoconus prediction index; TBUT: tear break-up time; OSS: ocular surface staining. ^†^ Chi-squared test.

**Table 3:** Comparison of the values of corneal topography before and after artificial tears instillation.

	Before Eye Drops	After Eye Drops	*p* Value
All (PSB + CMC, n = 135)			
Mean Sim K (D)	43.90 ± 2.02	44.07 ± 2.26	0.006 *
Astigmatism of Sim K (D)	1.38 ± 1.99	1.49 ± 2.03	0.095
Steep axis of astigmatism (°)	80.9 ± 43.6	83.1 ± 40.0	0.558
Changes in the axis of astigmatism ≥ 10° (n, %)	NA	70 (51.9%)	
Changes in the axis of astigmatism ≥ 30° (n, %)	NA	27 (20.0%)	
Central K of anterior axial curvature (D)	43.90 ± 2.03	43.80 ± 4.39	0.548
Mid K of anterior axial curvature (D)	43.32 ± 1.84	43.42 ± 1.88	0.027 *
Peripheral K of anterior axial curvature (D)	41.74 ± 1.66	41.78 ± 1.62	0.597
Mean total corneal power (D)	42.39 ± 4.20	42.77 ± 2.25	0.200
Astigmatism of total corneal power (D)	1.64 ± 2.56	1.79 ± 2.63	0.426
Kappa distance	0.37 ± 0.34	0.36 ± 0.30	0.659
KPI (%)	19.27 ± 21.06	17.53 ± 19.10	0.157
PSB (n = 101)			
Mean Sim K (D)	43.86 ± 2.04	44.04 ± 2.36	0.012 *
Astigmatism of Sim K (D)	1.31 ± 2.10	1.48 ± 2.17	0.049 *
Changes in the axis of astigmatism ≥ 10° (n, %)	NA	51 (50.5%)	
Changes in the axis of astigmatism ≥ 30° (n, %)	NA	22 (21.8%)	
Mean total corneal power (D)	42.26 ± 4.73	42.73 ± 2.34	0.238
Astigmatism of Total corneal power (D)	1.50 ± 2.36	1.83 ± 2.89	0.120
Kappa distance	0.38 ± 0.37	0.38 ± 0.32	0.992
KPI (%)	19.70 ± 20.67	16.70 ± 16.12	0.028 *
CMC (n = 34)			
Mean Sim K (D)	44.00 ± 1.97	44.17 ± 1.93	0.230
Astigmatism of Sim K (D)	1.58 ± 1.65	1.53 ± 1.54	0.608
Changes in the axis of astigmatism ≥ 10° (n, %)	NA	19 (55.9%)	
Changes in the axis of astigmatism ≥ 30° (n, %)	NA	5 (14.7%)	
Mean total corneal power (D)	42.78 ± 1.86	42.91 ± 1.98	0.278
Astigmatism of Total corneal power (D)	2.06 ± 3.08	1.68 ± 1.68	0.365
Kappa distance	0.34 ± 0.21	0.30 ± 0.20	0.282
KPI (%)	17.97 ± 22.44	19.99 ± 26.18	0.460

Values are presented as mean ± standard deviation. PSB: Polysorbate 80; CMC: 0.5% carboxymethylcellulose; Sim K: simulated keratometry; Sim Kastig: astigmatism between flat and steep; D: diopter; NA: non-available. * Statistically significant.

## Data Availability

The datasets used and/or analyzed in the current study are available from the corresponding author upon reasonable request.
